# Machine learning reveals ferroptosis features and a novel ferroptosis classifier in patients with sepsis

**DOI:** 10.1002/iid3.1279

**Published:** 2024-05-23

**Authors:** Zhigang Chen, Shiyou Wei, Zhize Yuan, Rui Chang, Xue Chen, Yu Fu, Wei Wu

**Affiliations:** ^1^ Department of Anesthesiology, Shanghai Pulmonary Hospital, School of Medicine Tongji University Shanghai China; ^2^ Department of Thoracic Surgery, Shanghai Pulmonary Hospital, School of Medicine Tongji University Shanghai China; ^3^ Medical Department, Shanghai Pulmonary Hospital, School of Medicine Tongji University Shanghai China

**Keywords:** biomarkers, diagnosis, ferroptosis, machine learning, sepsis

## Abstract

**Objective:**

Sepsis is an organ malfunction disease that may become fatal and is commonly accompanied by severe complications such as multiorgan dysfunction. Patients who are already hospitalized have a high risk of death due to sepsis. Even though early diagnosis is very important, the technology and clinical approaches that are now available are inadequate. Hence, there is an immediate necessity to investigate biological markers that are sensitive, specific, and reliable for the prompt detection of sepsis to reduce mortality and improve patient prognosis. Mounting research data indicate that ferroptosis contributes to the occurrence, development, and prevention of sepsis. However, the specific regulatory mechanism of ferroptosis remains to be elucidated. This research evaluated the expression profiles of ferroptosis‐related genes (FRGs) and the diagnostic significance of the ferroptosis‐related classifiers in sepsis.

**Methods and Results:**

We collected three peripheral blood data sets from septic patients, integrated the clinical examination data and mRNA expression profile of these patients, and identified 13 FRGs in sepsis through a co‐expression network and differential analysis. Then, an optimal classifier tool for sepsis was constructed by integrating a variety of machine learning algorithms. Two key genes, ATG16L1 and SRC, were shown to be shared between the algorithms, and thus were identified as the FRG signature of classifier. The tool exhibited satisfactory diagnostic efficiency in the training data set (AUC = 0.711) and two external verification data sets (AUC = 0.961; AUC = 0.913). In the rat cecal ligation puncture sepsis model, in vivo experiments verified the involvement of ATG16L1 and SRC in the early sepsis process.

**Conclusion:**

These findings confirm that FRGs may participate in the development of sepsis, the ferroptosis related classifiers can provide a basis for the development of new strategies for the early diagnosis of sepsis and the discovery of new potential therapeutic targets for life‐threatening infections.

## Graphical abstract

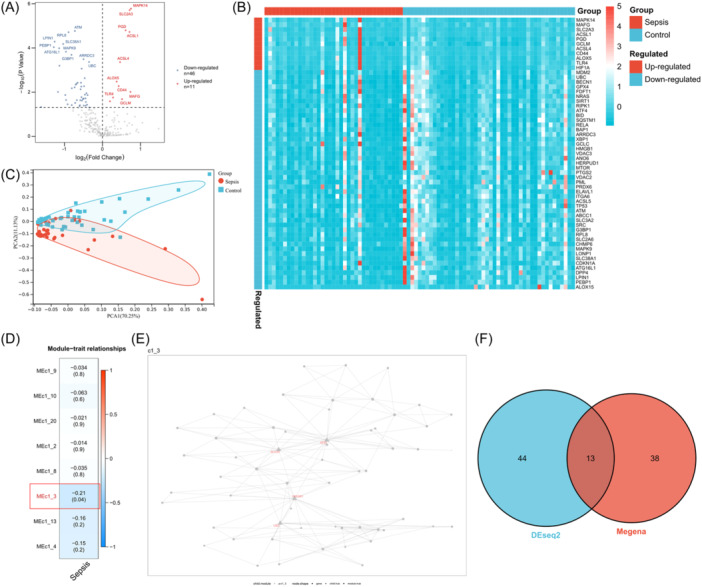

## 1 INTRODUCTION

Sepsis is an organ malfunction that may lead to death and is triggered when the host's normal response to inflammation is in a dysregulation state.^1^ Individuals who are hospitalized have a high mortality risk from sepsis.^2^ Although various methods for the management and treatment of sepsis have already been developed, the overall clinical results are not satisfactory.^3^ The host immune response during sepsis is highly heterogeneous. This hinders the early identification of patients with high mortality and their choice of targeted therapy.^4^ Furthermore, previous research has illustrated that early diagnosis is quite difficult due to a variety of complications associated with the disease and the lack of effective predictive tools.^5^ As a result, it is of the greatest priority to discover possible novel biological markers for the early detection of sepsis, with the goals of lowering death rates and improving patients' prognoses.

Ferroptosis is a mode of programmed cell death that relies on iron and is hallmarked by excessive accumulation of lipid hydroperoxides, thereby contributing to overwhelming lipid peroxidation and subsequent cell death.^6^, ^7^ The involvement of ferroptosis in cancer is an interesting issue since ferroptosis is a novel mode of cell death. Extensive research has demonstrated that inducing cancer cell death by ferroptosis provides a viable alternative to tumor treatment, particularly in malignant tumors that are resistant to traditional treatment.^8^ In recent years, ferroptosis has also been shown to be involved in various diseases and pathological processes.^9^, ^10^ Recently, it has been reported that ferroptosis regulated by Glutathione Peroxidase 4 (GPX4) may be a new pathophysiological mechanism of sepsis,^11^ that inhibits ferroptosis from playing a protective role in septic lung injury.^12^ Thus, exploring the contribution of ferroptosis in sepsis can be potentially useful. However, further research is needed to clarify the specific regulatory mechanism.

In this study, we integrated the clinical examination data and mRNA expression profile of septic patients and identified the characteristic genes expressed in sepsis through a co‐expression network and differential analysis. Then, the ferroptosis‐related genes (FRGs) in septic patients were detected by comparative analysis of the collected gene expression data. Lastly, based on the identified list of FRGs, a robust classifier was constructed for the diagnosis of septic patients by comparing a variety of machine learning algorithms, which were verified in two independent publicly available sepsis cohorts. And verified the role of the key genes by in vivo experiments. Taken together, our results can be used to develop new strategies for the early detection of sepsis.

## 2 METHODS

### 2.1 Data collection

To characterize the mRNA expression profile of septic patients, three data sets were extracted from the Gene Expression Omnibus (GEO) database: GSE6535, GSE63311, and GSE63042. These data sets include information gathered from septic patients' peripheral blood. The GSE63311 data set was obtained using the platform GPL10999 and the ratio of positive to negative results in this data set is more consistent with 1:1 (37:46), so we served it as a training data set for model development and variable identification.^13^ The GSE6535 and GSE63042 data sets were obtained from platform GPL4274 and platform GPL9115, respectively.^14^, ^15^ To eliminate batch effects, we externally validated the model using these data sets separately. All data sets had been Log2 standardized.

### 2.2 Identification of sepsis‐related ferroptosis characteristic genes

Differential analysis and co‐expression network were used to identify the characteristic ferroptosis gene in the peripheral blood of septic patients. FRGs were obtained from the database FerrDb^16^ (http://zhounan.org/ferrdb) and previously published studies.^6^, ^8^, ^17^, ^18^ Supporting Information: Table S1 summarizes the FRGs included in this research. First, the R package DEseq. 2 was used for differential expression analysis. To avoid omission, the threshold of FDR < 0.05 was applied to screen for differentially expressed ferroptosis genes (DFG), and the efficacy of DFGs was evaluated using principal component analysis (PCA). Subsequently, ferroptosis characteristics relevant to sepsis were determined by developing a co‐expression network in R utilizing a multiscale embedded gene co‐expression network analysis (MEGENA). This method showed better performance than traditional co‐expression network analysis.^19^ Briefly, according to the appropriate *K*‐value MEGENA can cluster the multidimensional data based on Planar Filtered Networks to obtain different modules. The intersection of DEseq. 2 and MEGENA results was considered to be the ferroptosis characteristic genes present in the septic disease. The functional gene enrichment analysis was conducted on the Metascape online platform (https://metascape.org/gp/index.html).^20^

### 2.3 Constructing a robust prediction model by a variety of machine learning approaches

The following R packages were utilized to develop machine learning models: XGBoost, e1071, Random Forest, Boruta, rms, and glmnet.^21^, ^22^, ^23^, ^24^ The most important ferroptosis features were first screened out using the extreme gradient boosting (XGBoost), Random Forest and Boruta (RFB), support vector machine (SVM), and least absolute shrinkage and selection operator (LASSO) regression analyses from the whole data set. The common gene between LASSO, SVM, RFB, and XGBoost analysis was determined to be the most important sepsis‐related ferroptosis feature and was employed for subsequent construction and training of the prediction model. Then, the effectiveness of each machine learning classifier in the data set was evaluated by fivefold cross‐validation. To be more precise, the data set GSE63311 was divided into five equal parts, and then in each of the five iterations, 4/5 of the data set was included in the training data so that the predictive model could be trained. Next, after training the model, it was applied to the remaining 1/5 of the training data. The outcomes of the predictions made by each of the five iterations were integrated and the effectiveness of the classifier was evaluated by constructing receiver operating characteristic (ROC) curves, decision curves, and confusion matrices. Finally, XGBoost was regarded as the best classifier to be the prediction model of sepsis and was applied to two external validation queues to evaluate the generalization capability of the prediction model.

### 2.4 Animal experiment

All animal experiments were approved by the Institutional Animal Care and Use Committee of Shanghai Pulmonary Hospital (No: K23‐318) and following the Guide for the Care and Use of Laboratory Animals published by the NIH. 30 male Sprague‐Dawley (SD) rats at 6–8 weeks of age were purchased from Vital River Laboratory Animal Technology Company. All rats were kept in a specific pathogen‐free animal laboratory, temperature control, 12/12 h light/dark cycle, free access to food and water. Cecal ligation and puncture (CLP) sepsis model was established with reference to previous studies.^25^ Subsequently, the skin, muscle layer, and peritoneum were cut layer by layer below the midpoint of the rat abdominal white line. The abdominal cavity was explored directly, and the terminal cecum was removed and placed outside the abdominal wall. One end of the cecum was ligated and punctured twice with a 21‐gauge needle. After squeezing out the cecum contents, the cecum was reduced, and the incision was closed in the muscle and skin layers. The anesthesia period of animals is not yet over, and it is important to keep them warm (37°C), raise them in single cages, and replace the bedding to keep their skin dry. Animals should not be given any food or drink before they are fully awake after surgery. After they are awake, they can be given water first, followed by food.

Animal experiments follow the 3R (replacement, reduction, refinement) principle. After adapting to the environment, healthy rats were included in the experiment. If the rat did not respond to gentle stimulation, lost significant weight, was unable to eat or drink, moved slowly and sluggishly, was visibly anxious and irritable, or shown other signs of distress, the experiment should be excluded. Grouping is conducted using a simple randomization method. Based on the methods of similar study,^26^ the sample size for each group was six rats. Thirty aged male SD rats were randomly divided into five groups: Naive group (*n* = 6), sham operation group; Sepsis Group (*n* = 6), a rat model of sepsis induced by CLP; The Sepsis + Vector group (*n* = 6), the AAV 9 empty vector was injected into the tail vein and lateral ventricle 1 h after CLP; The Sepsis + ATG16L1 group (*n* = 6), AAV 9‐ATG16L1 overexpression vector was injected into the tail vein and lateral ventricle 1 h after CLP; The Sepsis + SRC group (*n* = 6), AAV 9‐SRC overexpression vector was injected into the tail vein and lateral ventricles 1 h after completion of CLP. All the experiment rats were killed at 24 h after CLP or sham surgery. All rats in this study met the inclusion criteria, so no rats were excluded. When the rats were dying or unable to move, they will be euthanized by injecting excessive sodium pentobarbital into their abdominal cavity. This study conducted randomized grouping, blind data measurement and analysis, and followed the principle of control to reduce potential confounding factors. Only Zhigang Chen was aware of the group allocation at the different stages of the experiment.

### 2.5 qRT‐PCR

Total RNA extraction was performed using the Trizol (ThermoFisher) reagent. Real‐time PCR amplification was performed on a Applied Biosystems 7500 real‐time PCR system using the SuperScript™ III Platinum™ SYBR™ Green one‐step qRT‐PCR kit (ThermoFisher) according to the manufacturer's instructions. The relative expression of the target genes was measured by the 2−ΔΔCt method, with GAPDH serving as an internal reference.

### 2.6 ELISA

0.5 mL of rat blood was centrifuged at 3500 r/min for 10 min and the supernatant was frozen for ELISA. Let the ELISA kit stand at room temperature for 30 min, diluted the standard according to the required concentration, added 100 μL of serum and standard to each well, added universal diluent and standard to the blank well, 36°C for 90 min. After washing the plate, 100 μL of biotinylated antibody was added to each reaction well, and the antibody dilution solution was added to the blank well for 60 min. After washing the plate, 100 μL of the enzyme standard antibody was added to each reaction well, and the enzyme binding substance dilution solution was added to the blank well for 30 min. After washing the plate, 100 μL of 3,3′, 5,5′‐tetramethylbenzidine was added to each well for 15 min. Subsequently, 100 μL of termination solution was added, the absorbance (A value) was read by the microplate reader within 3 min, and the standard curve was drawn according to the concentration of the standard and the corresponding A value.

### 2.7 Histological staining

After the rats were euthanized, the brain, lung, and myocardial tissue of the rats were extracted, embedded in paraffin, and made into 5 μm thick sections, dewaxed and rehydrated. Altered tissue architecture in rat tissues were detected using HE staining, neuronal activity in rat brain tissue using Nissl staining, and the proportion of apoptotic cells in rat myocardial and lung tissues using TUNEL staining.

### 2.8 Statistical analysis

Experimental correlation statistical analysis was performed using the Graphpad Prism8.02 software. Data from experiments was expressed as mean ± SD. *T*‐test was used to compare differences between two groups. Comparisons between multiple groups were performed using ANOVA with Tukey as a post hoc test. Pearson's correlation analysis was used to detect the correlation between the two gene expressions in GSE63311, *p < .05 was considered statistically significant.

## 3 RESULTS

### 3.1 Identification of ferroptosis characteristic genes in the peripheral blood of septic patients

**Figure 1:**
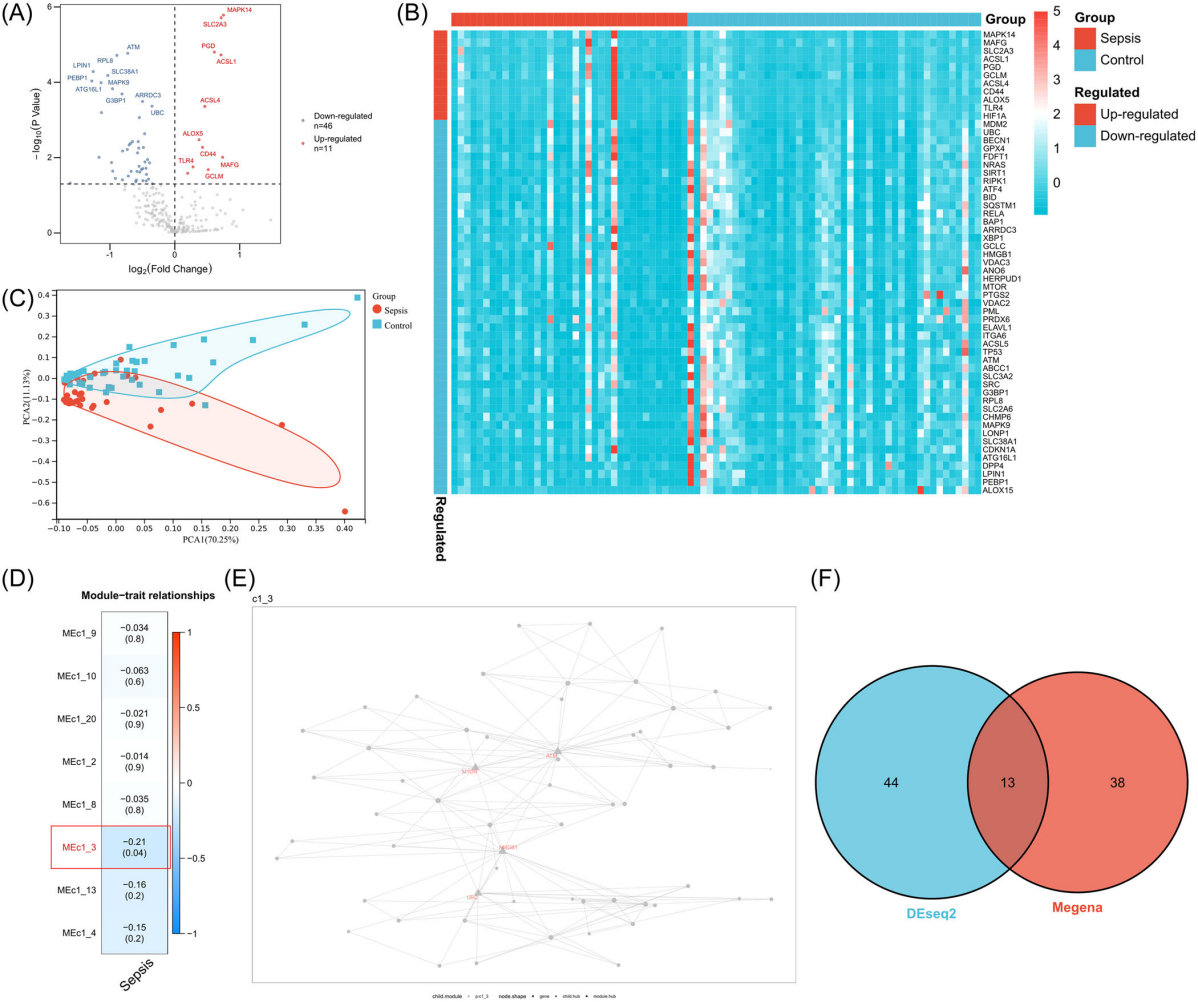
Identification of ferroptosis‐associated genes in septic patients' peripheral blood. (A) Differentially expressed genes (DEGs) between the septic and control groups are depicted on a volcano map (FDR < 0.05). Red and blue colors denote the upregulated and downregulated genes, correspondingly. (B) Heat map showing the transcription map of significant DEGs. (C) Principal component analysis of DEGs shows good discrimination ability. (D) MEGENA shows the heat map of the correlation between gene module and sepsis. The most relevant module is marked with a red box. (E) Module C1_3 gene link networks. (F) Venn diagram showing the intersection of differential analysis and MEGENA results. Thirteen ferroptosis characteristic genes were identified.

First, DFGs were identified in the peripheral blood of septic and control individuals using differential analysis. To avoid omission, the threshold was set to FC > 1 and *p* < .05. There were 57 DFGs found, 46 of which were downregulated and 11 were upregulated (Figure 1A). The heat map shows the transcription map of DFGs (Figure 1B). Our PCA results showed that the identified differentially expressed genes could distinguish septic from control samples well (Figure 1C).

Subsequently, a MEGENA network was constructed to identify sepsis‐related ferroptosis genes, and *k* = 4 was selected to achieve the minimum error (Supporting Information: Figure S1A). The heat map showed that the network could be well divided into four categories (Supporting Information: Figure S1B). Based on a threshold of *k* = 4, a total of eight network modules were identified (Supporting Information: Figure S1C). Module C1_3 exhibited the strongest correlation with sepsis (*r* = −.21, *p* = .04) (Figure 1D). The subnetwork C1_3 is shown in Figure 1E. This group contained 51 module genes, among which MTDH, NRAS, PRDX1, and VDAC3 were identified to be hub genes. Finally, the results of the differential analysis and MEGENA were integrated further identifying a total of 13 ferroptosis characteristic genes (Figure 1F).

**Figure 2:**
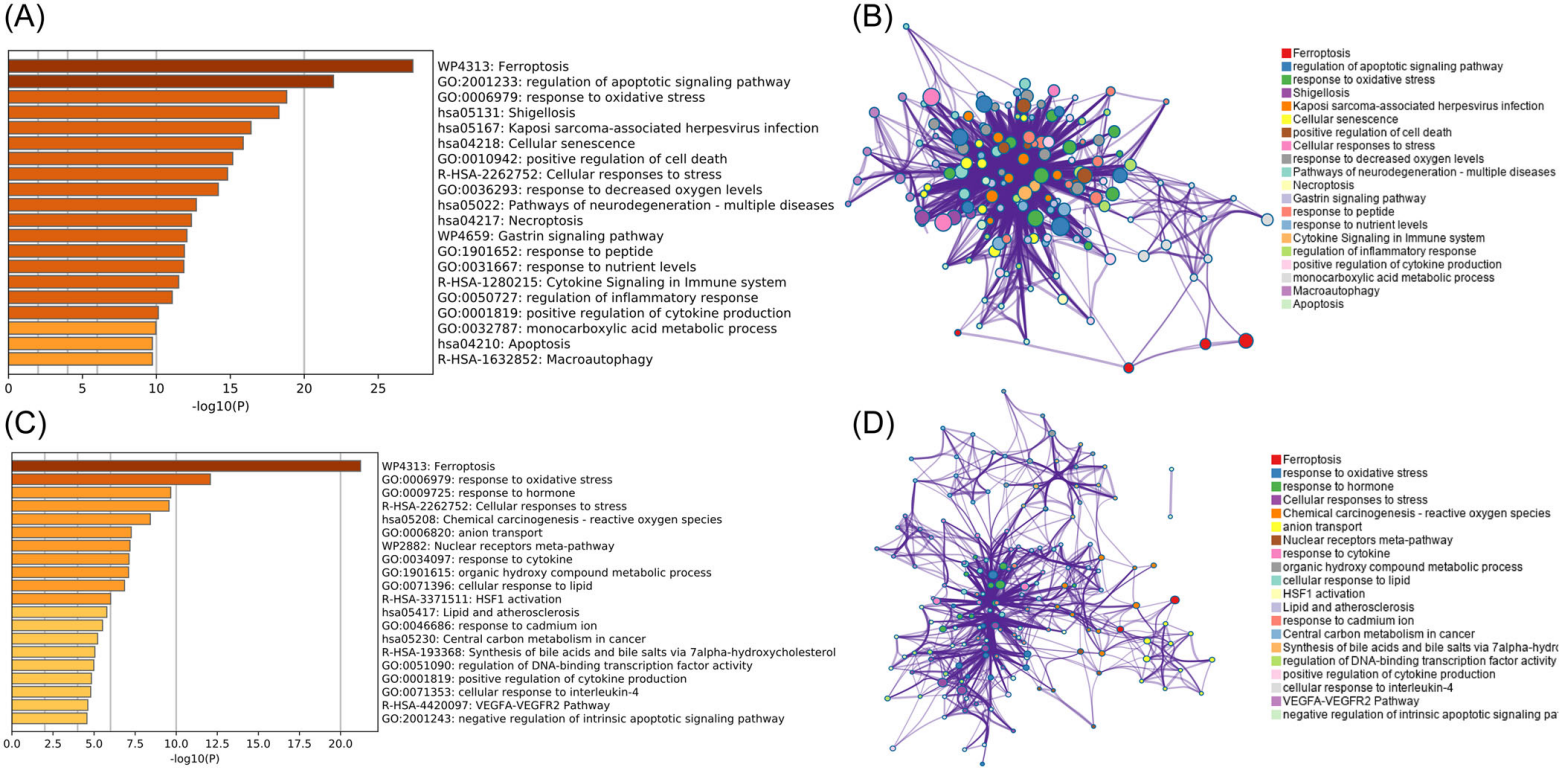
Functional enrichment analysis of ferroptosis characteristic genes. (A) Differentially expressed ferroptosis genes (DFG)‐related functional enrichment analysis findings (B) Biological function network diagram of DFGs. (C) Functional enrichment results of C1_3 module genes. (D) Biological function network diagram of C1_3 module genes.

Moreover, Metascape was used to analyze the functional enrichment of DFGs and module genes to explore their biological functions. The results showed that DFGs were mainly involved in oxidative stress, apoptosis, and cytokine pathways (Figure 2A). The functional network of DFGs is shown in Figure 2B. Similarly, module genes included in C1_3 were mainly related to cytokine pathway, oxidative stress, and metabolism‐related pathway (Figure 2C). The functional network of C1_3 module genes is shown in Figure 2D.

### 3.2 Correlation analysis of genes associated with ferroptosis

**Figure 3:**
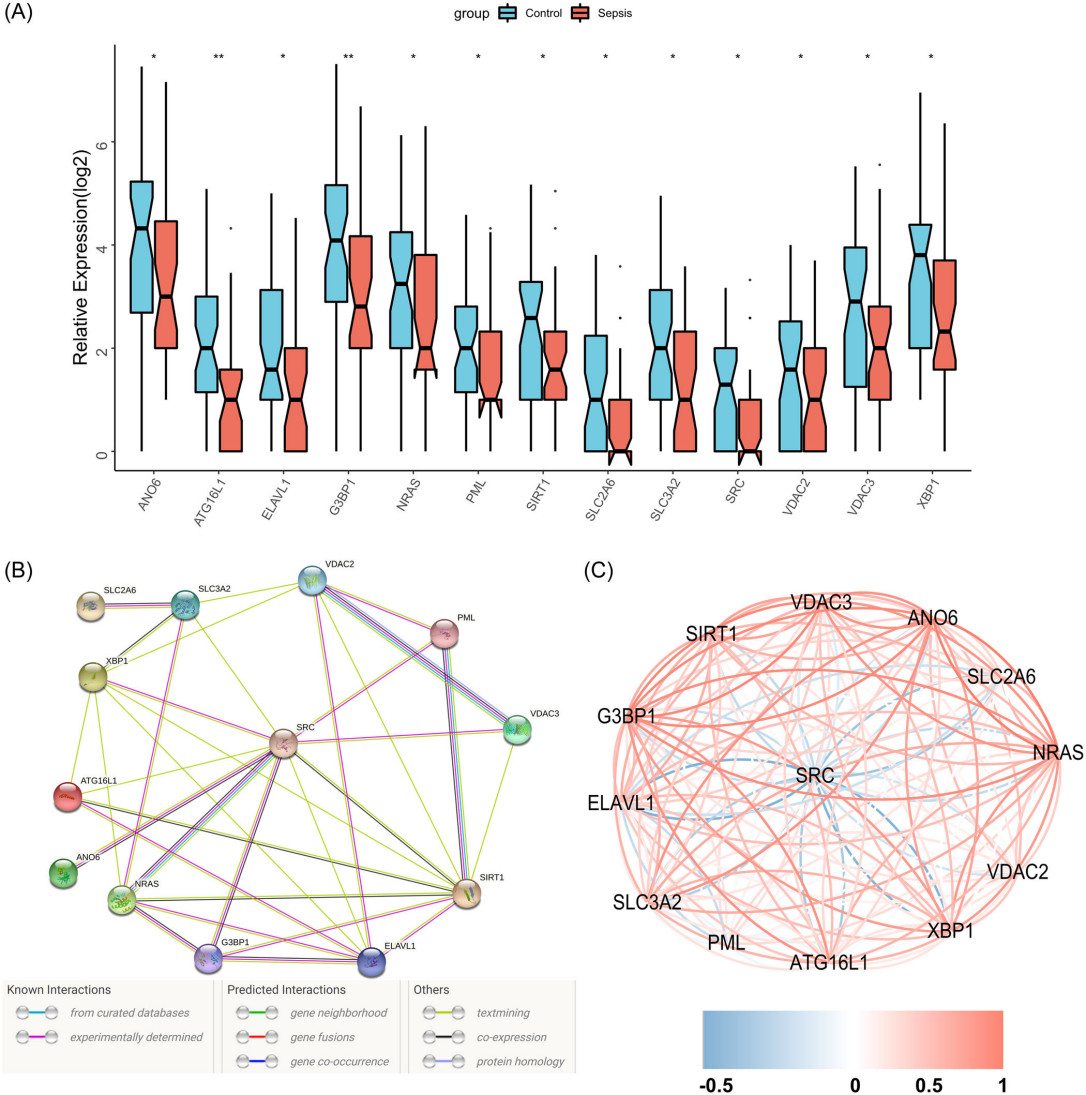
Co‐expression network of ferroptosis characteristic genes. (A) Box diagram illustrating the differential expression of ferroptosis characteristic genes between septic and the control group. Wilcox test, *p < .05; **p < .01. (B) Protein–protein interaction network of ferroptosis characteristic proteins. (C) The correlation network of ferroptosis characteristic genes. Positive and negative correlations are denoted by the red and blue colors, correspondingly, which only shows the result with *p* < .05 significance.

There was a substantial downregulation in the expression of all 13 FRGs in sepsis samples relative to controls (Figure 3A). The protein–protein interaction networks of the proteins coded by the 13 genes were obtained using the String database, in which SRC is the protein hub of the network (Figure 3B). Figure 3C shows the correlation network of the 13 genes indicating pairs with significant correlation (*p* < .05). The findings illustrated that most of the ferroptosis characteristic genes were positively correlated with each other, while *SRC* was negatively correlated with other genes.

### 3.3 Constructing and validating the best ferroptosis‐related sepsis classifier tool

**Figure 4:**
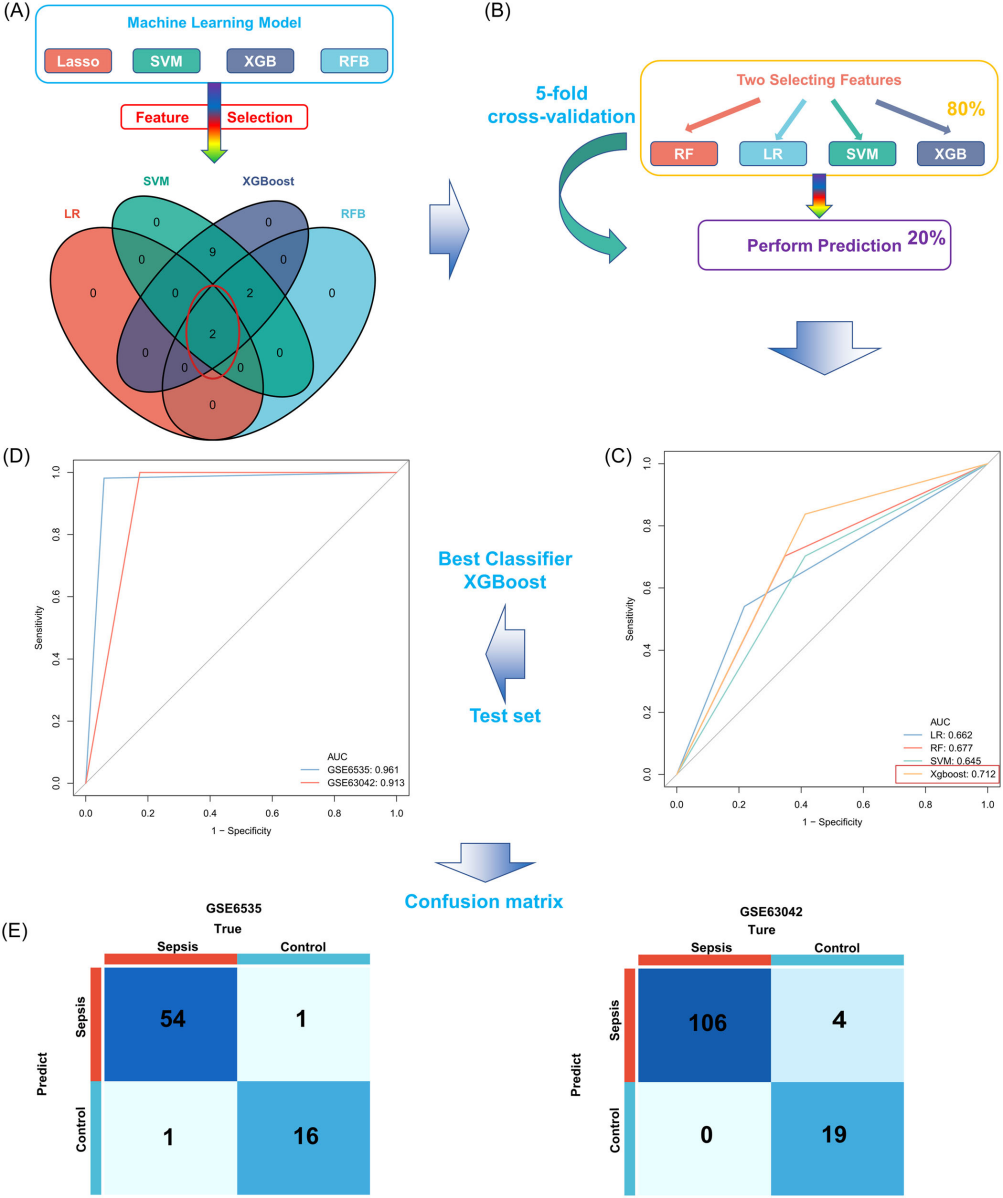
Development and verification of sepsis predictor related to ferroptosis. (A) In the data set GSE63311, the two most important ferroptosis characteristics were selected based on four machine learning algorithms. (B) A diagrammatic scheme of the four machine learning algorithms used in training and verifying stable classifiers through fivefold cross‐validation using the training set; (C) Comparison of the four predictors' receiver operating characteristic (ROC) curves derived from a cross‐validation analysis based on the training set. (D) Application of the best classifier, XGBoost, to the ROC curves of the two external verification data sets. (E) Confusion matrix of the predictor in two external validation data sets. Left, GSE6535; Right, GSE63042.

Four mature machine learning methods, including LASSO, RFB, SVM, and XGBoost, were used to identify the most critical ferroptosis signatures in the training set. From our analysis, 2, 4, 13, and 13 genes were identified, respectively (Supporting Information: Figure S2), detailed gene list of each algorithm is provided in Supporting Information: Table S2. Two key genes, *ATG16L1* and *SRC*, were shown to be shared between the four algorithms, and thus were identified as the FRG signature of classifier (Figure 4A). Subsequently, four supervised machine learning algorithms, LASSO (nfold = 5, type.measure = “class”), SVM (number = 20), RF (doTrace = 2, ntree = 1000, maxRuns = 100), and XGBoost (max_depth = 2, eta = 1, silent = 1, nround = 25), were applied to evaluate the utility of these gene signatures based on ROC curves through a fivefold cross‐validation analysis (Figure 4B). It was found that the classifier trained based on two key ferroptosis signatures could distinguish septic patients well (LR, AUC = 0.662; RF, AUC = 0.677; SVM, AUC = 0.645; XGBoost, AUC = 0.712; Figure 4C). We also used decision curve to evaluate the clinical decision benefits of each models. As shown in Supporting Information: Figure S3, the Xgboost model had the best decision‐making benefits. It is worth noting that the XGBoost model showed the greatest AUC value. Next, the results recorded from a comprehensive analysis of the four algorithms' performance are described in Table 1. The LR model achieved the largest Kolmogorov–Smirnov (KS) value (KS = 0.442), followed by the XGBoost model (KS = 0.425). The LR model showed a leading advantage in distinguishing the two groups of samples; however, the XGBoost model had the best accuracy (0.699). As a serious emergency, more accurate identification of septic patients is of prime importance. Since false positives are acceptable, recalls should be considered equally important. Accordingly, the XGBoost model performed the best, achieving the highest recall (0.838). In contrast with the XGBoost model, the LR model had lower accuracy and recall.

**Table 1:** Evaluation results of four machine learning classifier.

Model	TP	TN	FP	FN	Precision	Recall	F1 score	Accuracy	KS	Error
LR	20	36	10	17	0.667	0.541	0.597	0.675	0.442	0.325
RF	26	30	16	11	0.619	0.703	0.658	0.675	0.360	0.325
SVM	26	27	19	11	0.578	0.703	0.634	0.639	0.333	0.361
Xgboost	31	27	19	6	0.620	0.838	0.713	0.699	0.425	0.301

Abbreviations: FN, False negative; FP, False positive; KS, Kolmogorov–Smirnov; TN, True negative; TP, Ture positive.

Based on our analysis, the XGBoost model was chosen to further verify the performance of the developed predictive tool using two external data sets. Surprisingly, the model showed excellent performance in our external verification. The ROC curves showed an AUC = 0.961 for GSE6535 and AUC = 0.913 for GSE63042 (Figure 4D). In addition, the effectiveness of the confusion matrix visual classification model was also evaluated (Figure 4E). It is worth noting that in the GSE6535 data set, the classifier had a satisfactory performance, with errors only recognized in two patients. Consistently, the classifier also showed an excellent performance using another data set, GSE63042. All 106 septic patients and 19 healthy subjects were correctly identified, and only 4 healthy subjects were incorrectly identified as having sepsis. However, the two data sets had few healthy controls, which may introduce a bias in determining the effectiveness of the classifier.

### 3.4 ATG16L1 and SRC have significantly low expression in organ damage caused by sepsis

**Figure 5:**
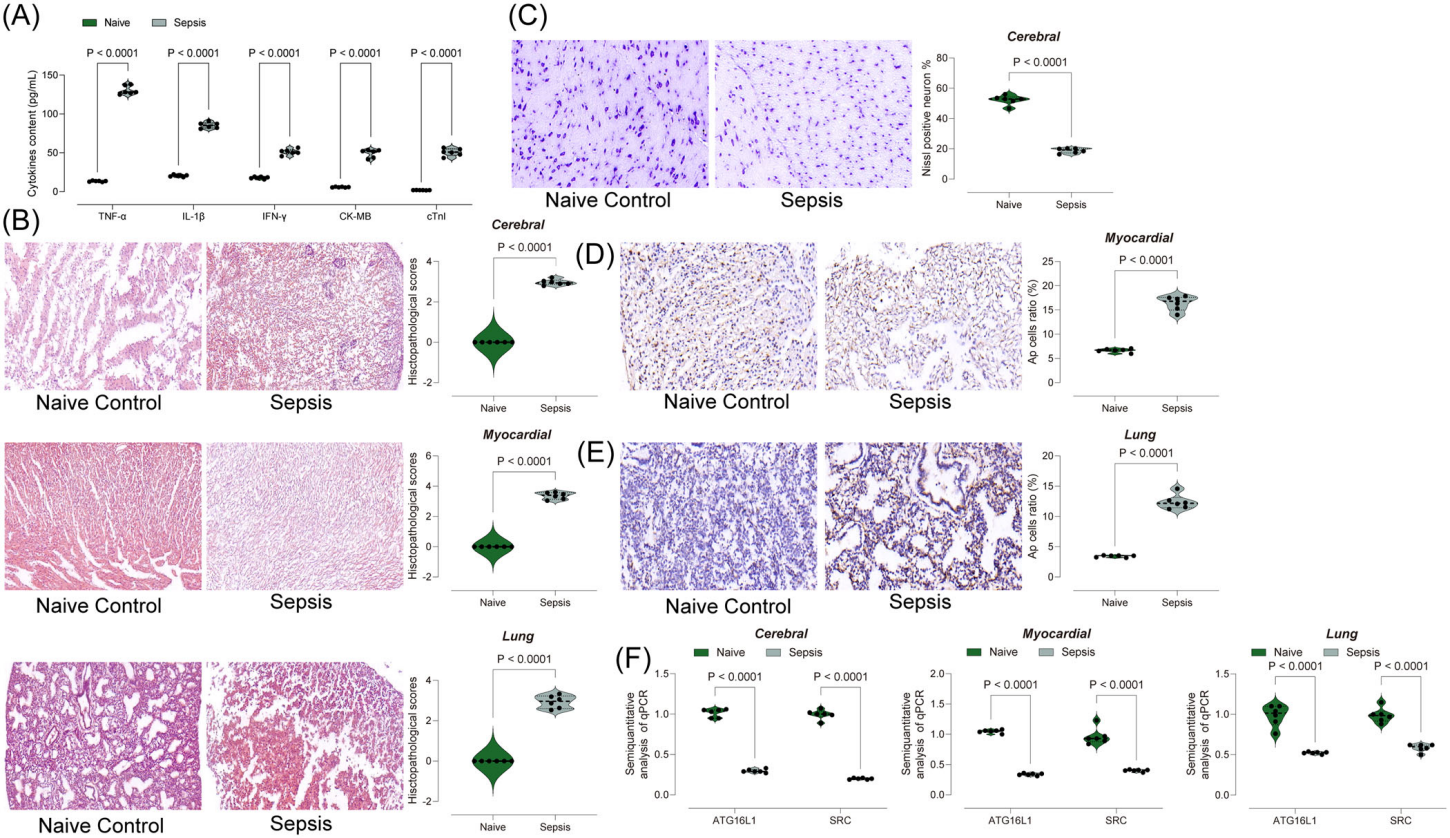
ATG16L1 and SRC showed significantly low expression in lung, myocardial, and brain tissues in Sepsis rats. (A) ELISA for detecting the content of IFN‐gamma, IL‐1beta, CK‐MB, cTNI, and TNF‐alpha in the serum of rats; (B) HE staining showed the histological structure of lung tissue, myocardial tissue and brain tissue in rats; (C) Nissl staining indicates the activity of neurons in the rat brain tissue; (D) and (E) TUNEL staining indicates the proportion of apoptotic cells in rat myocardial and lung tissues; (F) qPCR shows the mRNA expression levels of ATG16L1 and SRC in rat myocardial, brain and lung tissues. Each group contained six rats.

In Figure 3, we found that ATG16L1 and SRC, genes associated with ferrotosis, had significantly decreased expression in sepsis. Previous studies showed that miR‐223 could promote inflammatory damage in the CNS system by targeting ATG16L1,^27^ and that ATG16L1 has been shown to inhibit lung injury caused by infection by regulating the expression of IL‐22 or IFN.^28^ In our prediction, SRC was found to be significantly low expressed in the tissues of sepsis patients, but previous studies believed that SRC could lead to inflammatory response through activation of immune cells, which contradict the results of our bioinformatics analysis. However, we believed that the expression level of SRC can be used as a basis for early diagnosis of sepsis. Therefore, to test our conjecture, a rat sepsis model was established by CLP surgery. ELISA was used to confirm the significant increase of inflammation in septic rats (Figure 5A). HE staining showed obvious structural disorder, blurred cell boundary and increased immune cell infiltration of lung and myocardial tissue in septic rats (Figure 5B). Moreover, Nissl staining showed a significant reduction in neuronal activity in septic brain tissue (Figure 5C), TUNEL staining showed that the number of apoptosis in lung and myocardial tissue of septic rats increased significantly (Figure 5D,E), and qPCR results showed that ATG16L1 and SRC expression in septic rats was significantly reduced (Figure 5F).

### 3.5 Overexpression of ATG16L1 or SRC impaired organ damage in sepsis

**Figure 6:**
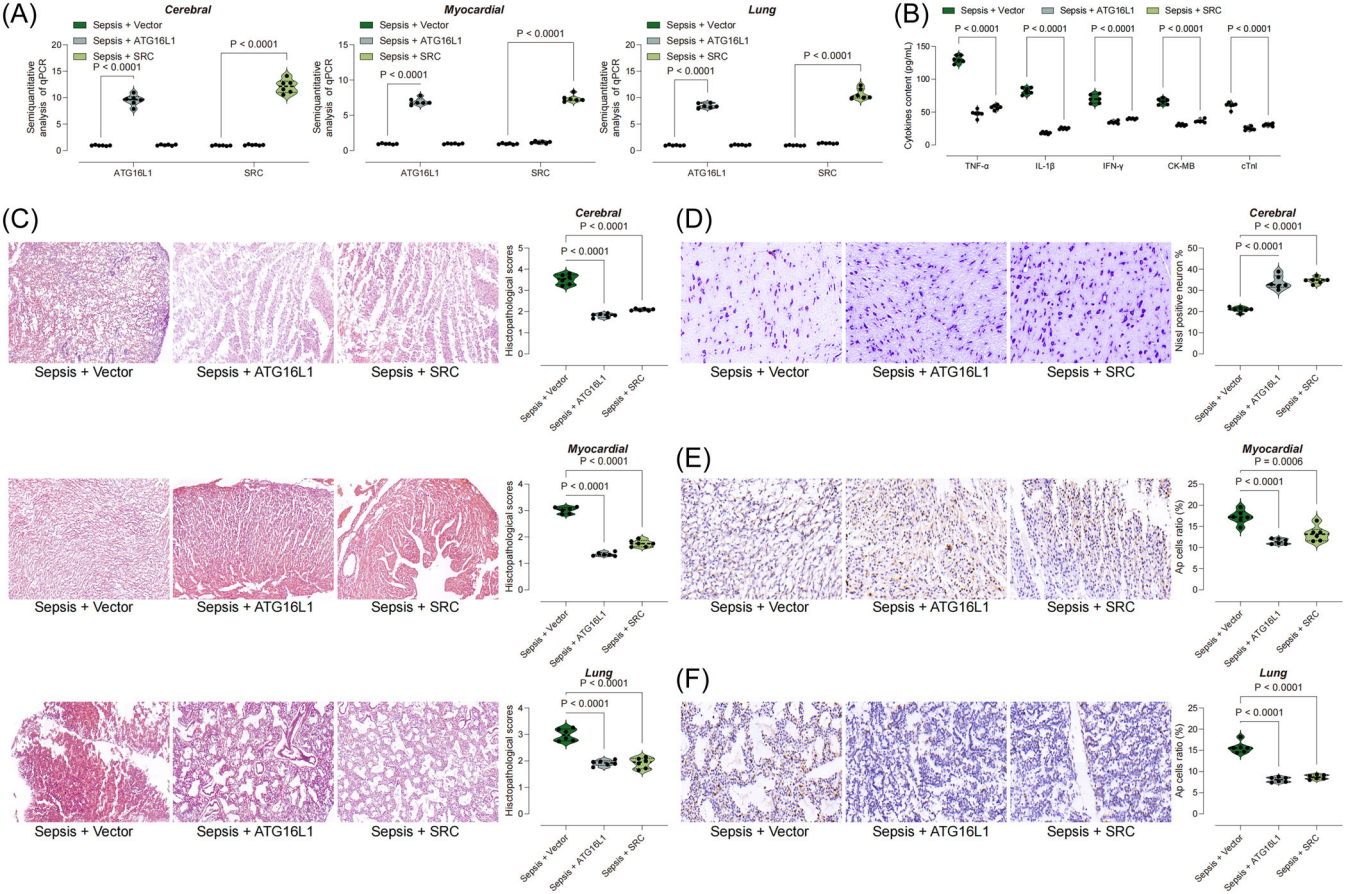
Overexpression of ATG16L1 or SRC impaired organ damage in sepsis. (A) qPCR reflects the mRNA expression levels of ATG16L1 and SRC in rat myocardial, brain and lung; (B) ELISA detection of IFN‐gamma, IL‐1beta, CK‐MB, cTNI, TNI and TNF‐alpha in rat serum; (C) HE staining to detect the histological structure of rat myocardial tissue, brain tissue and lung tissue changes; (D) Nissl staining shows the activity of neurons in the rat brain tissue; (E) and (F) TUNEL staining showing the proportion of apoptotic cells in rat myocardial and lung tissues. Each group contained six rats.

To further verify the role of ATG16L1 and SRC in organ damage in septic rats, we constructed the AAV 9 overexpression vector for ATG16L1 and SRC, which was injected into septic rats through the tail vein or into the lateral ventricle. First, the mRNA expression levels of ATG16L1 and SRC in myocardial, lung and brain tissues were measured by qPCR to confirm successful overexpression (Figure 6A). Subsequently, we found that after the overexpression of ATG16L1 and SRC, the inflammatory response in septic rats was significantly attenuated (Figure 6B), and the pathological results in myocardial tissue, brain tissue and lung tissue were significantly repaired (Figure 6C). Both Nissl and TUNEL staining indicated that overexpression of ATG16L1 and SRC significantly promoted the recovery of neuronal activity after sepsis and inhibited apoptosis in myocardial and lung tissues of septic rats (Figure 6D–F).

## 4 DISCUSSIONS

Sepsis remains an important public health challenge. Sepsis is a fatal organ failure triggered by the dysregulated host response to infection, which contributes significantly to patient deaths.^2^, ^29^ Despite its high incidence rate and mortality rate, only a few drugs help treat sepsis. Many efforts have been made to achieve personalized treatment strategies for sepsis,^30^, ^31^ however, these studies focused on routinely collected clinical data. Although these characteristics are easy to obtain under limited resources, they do not capture important pathophysiological changes and reveal potential underlying mechanisms. Genomic changes in septic patients may provide important prognostic and predictive information,^32^ which is expected to be used in developing effective new biomarkers to augment the early diagnosis, monitoring, and treatment intervention for sepsis.

In this study, the characteristic ferroptosis genes in the peripheral blood of septic patients were screened using MEGENA and differential analysis. A stable sepsis diagnostic model integrating the two most important ferroptosis markers, *ATG16L1* and *SRC*, was constructed using a variety of machine learning models. Additionally, two external data sets were used to verify the diagnostic model's efficiency in making predictions. The involvement in the early course of sepsis was verified in vivo.

To develop a reliable sepsis diagnosis model, the mRNA expression profile of septic patients was first analyzed, leading to the identification of 57 DFGs. Compared with other co‐expression network algorithms, MEGENA has been shown to have a better performance.^33^ Therefore, we used this tool to identify the gene module most related to sepsis, which includes 51 characteristic ferroptosis genes. These genes were shown to be correlated with cytokine and oxidative stress‐related pathways via functional annotation. Increasing evidence shows that oxidative stress is the cornerstone in the pathogenesis of sepsis. It has been shown that oxidative stress causes impaired vascular permeability, decreased cardiac function, mitochondrial dysfunction, and impaired respiratory function, among others.^34^ In addition, oxidative stress has been shown to alter the state of epithelial cells and promote pro‐inflammatory, procoagulant, and adhesive cellular processes.^35^ Moreover, cytokines were observed to increase in a variety of intensive care syndromes, such as sepsis and acute respiratory distress syndrome, and the release of inflammatory cytokines would induce the production and subsequent release of new cytokines, causing a cytokine storm.^36^ One possible explanation for the wide variety of local and remote signs is a cytokine cascade or cytokine storm, which eventually exceeds its limit, resulting in cell and organ damage.^37^

Ferroptosis plays an active role in regulating immune function in an inflammatory environment.^38^, ^39^ In recent years, new views believe that ferroptosis has broad prospects in the occurrence, development, and prevention of sepsis.^11^, ^12^ Therefore, the utility of FRGs as potential biomarkers was explored in this study. The analysis results of MEGENA and DFGs were combined to find the characteristic ferroptosis genes in the peripheral blood of septic patients. Taking into account the time and cost savings for therapeutic use, four mature machine learning models were evaluated, XGBoost, SVM, RFB, and LASSO, and a diagnostic model composed of two ferroptosis characteristic genes, *ATG16L1* and *SRC*, was proposed. *ATG16L1* is involved in the regulation of autophagy in ferroptosis. It has been reported that mice lacking *ATG16L1* had accelerated LPS (Lipopolysaccharide) ‐mediated necroptosis and sepsis.^40^ In addition, mice lacking *ATG16L1* in bone marrow cells are more likely to die of LPS‐mediated sepsis.^41^ Furthermore, *ATG16L1* plays a protective role in the process of infection. Our findings suggest that the decrease of *ATG16L1* in septic patients might lead to a more serious infection. Therefore, *ATG16L1* may be a potential diagnostic and therapeutic target for sepsis.

On the other hand, it has been demonstrated that SRC performs a significant role in modulating the recruitment of immune cells and host defense.^42^ Recent studies have reported that regulating *SRC* activation can improve excessive inflammation and immune collapse in sepsis.^43^ Further, some studies have also shown that inhibiting *SRC* activity can reduce acute lung injury and acute renal injury secondary to sepsis.^44^, ^45^ Our findings show that *SRC* expression was reduced in septic patients. This might be used as a basis for sepsis‐related early diagnostic techniques. However, as previously stated, upregulation or inhibition of SRC expression have both been shown to reduce tissue damage caused by excessive inflammation. Appropriate SRC concentrations need to be further explored, the two sides of *SRC* should also be taken into account to formulate new molecular treatment strategies.

Machine learning has broad applications in the field of biomedicine and shows outstanding performance in clinical diagnosis and precision treatment.^46^ In this study, the LR, RF, SVM, and XGBoost classifiers were evaluated. Upon comprehensively evaluating the prediction ability of the classifier, a stable sepsis classifier based on XGBoost was constructed. The tool showed excellent prediction ability in the training set (AUC = 0.712, Accuracy = 0.699). Moreover, excellent performance was also achieved using two external validation queues (AUC = 0.961 and 0.913). These findings suggest the potential of using predictive tools similar to our model for the early diagnosis of sepsis. More importantly, considering that sepsis is clinically severe, false positives were acceptable when identifying patients with sepsis. Moreover, our model showed excellent recall in all three cohorts, which indicates the low possibility of missed diagnosis. In addition, the two gene diagnostic models were also convenient for clinical operation. Therefore, we suggest that our sepsis classifier model has a high clinical application value.

In vivo experiments in a rat sepsis model validated the differential expression of ATG16L1 and SRC. After regulating the overexpression of specific targets, it could significantly alleviate the degree of organ damage in septic rats, further indicating that ATG16L1 and SRC function early in sepsis, and the increased expression was able to relieve sepsis.

Although our findings may provide a framework for future research, we recognize several deficiencies in this study. First, since we were not able to extract the clinical information of individuals in the public data sets, the diagnostic efficacy of theclassifier was not compared with the traditional infection indicators used in clinics, such as CRP, which reduces the reliability of our results. Different ages may also contribute to the high prevalence of sepsis, so age‐related subgroup analyses are also necessary. Unfortunately, we were unable to obtain age information for the publicly available data set, and further studies are needed in the future. Second, the number of each subgroup of the external validation data is very unbalanced, which may have led to the model having a falsely high accuracy rate in the external validation. Higher‐quality data are needed for further model validation. In addition, considering the dual biological functions of the two ferroptosis characteristic genes, *ATG16L1* and *SRC*, it is necessary to elucidate their exact role and specific regulatory mechanisms in sepsis through in vivo and in vitro studies. Future studies should focus on large clinical cohorts to encompass as many patients with different characteristics as possible, with exhaustive follow‐up to add information about the predictive efficacy of iron death classifiers in different clinical subgroups.

## 5 CONCLUSION

In conclusion, we were able to integrate the results of MEGENA and differential analysis of septic patients and identify the important ferroptosis characteristic genes in sepsis using different machine learning algorithms. Based on these genes, a robust sepsis diagnostic model was constructed, which showed satisfactory performance in the training and test sets. Collectively, our findings may offer a foundation for the development of new strategies for the early diagnosis of sepsis and the discovery of new potential therapeutic targets for life‐threatening infections.

## AUTHOR CONTRIBUTIONS

Zhigang Chen and Wei Wu proposed the study conception. Zhigang Chen, Rui Chang, and Shiyou Wei performed the data cleaning. All authors participated in the analysis of the data, with Zhize yuan, Wei Wu, and Zhigang Chen involved in the statistical selection and quality control. Zhigang Chen, Yu Fu, and Zhize yuan finished the experimental validation. Zhigang Chen, Rui Chang, and Shiyou Wei prepared the figures and tables. Zhigang Chen, Yu Fu, Shiyou Wei, and Rui Chang wrote the paper, and Wei Wu, Zhigang Chen, and Zhize yuan reviewed the final manuscript.

## CONFLICT OF INTEREST STATEMENT

The authors declare no conflict of interest.

## ETHICS STATEMENT

All animal experiments were approved by the Institutional Animal Care and Use Committee of Shanghai Pulmonary Hospital (No: K23‐318).

## Supporting information

Supporting information.

Supporting information.

Supporting information.

## Data Availability

The data sets used and/or analyzed during the current study are available from the corresponding author on reasonable request.
