# Supplementary material for: Machine learning reveals ferroptosis features and a novel ferroptosis classifier in patients with sepsis
Source: Immun Inflamm Dis. 2024 May 23;12(5):e1279. doi: 10.1002/iid3.1279 (PMC11112629; doi:10.1002/iid3.1279)
Supplement: Supplementary file 1 — Supporting information. [file IID3-12-e1279-s001.docx]

| **Table S1. The ferroptosis genes** | | | | | | |
| --- | --- | --- | --- | --- | --- | --- |
| ABCC1 | BECN1 | ENPP2 | HSD17B11 | MIR212 | PRDX6 | SQSTM1 |
| ACACA | BID | EPAS1 | HSF1 | MIR30B | PRKAA1 | SRC |
| ACO1 | BLOC1S5-TXNDC5 | FADS2 | HSPA5 | MIR4715 | PRKAA2 | SRXN1 |
| ACSF2 | BNIP3 | FANCD2 | HSPB1 | MIR6852 | PRNP | STAT3 |
| ACSL1 | BRD4 | FBXW7 | IDH1 | MIR9-1 | PROM2 | STEAP3 |
| ACSL3 | CA9 | FDFT1 | IFNG | MIR9-2 | PSAT1 | STMN1 |
| ACSL4 | CAPG | Fer1HCH | IL33 | MIR9-3 | PTGS2 | TAZ |
| ACSL5 | CARS1 | FH | IL6 | MT1G | RB1 | TF |
| ACSL6 | CAV1 | FLT3 | IREB2 | MT3 | RELA | TFAP2C |
| ACVR1B | CBS | FTH1 | ISCU | MTDH | RGS4 | TFR2 |
| AGPAT3 | CD44 | FTL | ITGA6 | MTOR | RIPK1 | TFRC |
| AIFM2 | CDKN1A | FTMT | JDP2 | MUC1 | RPL8 | TGFBR1 |
| AKR1C1 | CDKN2A | G3BP1 | JUN | MYB | RRM2 | TLR4 |
| AKR1C2 | CDO1 | G6PD | KEAP1 | MYC | SAT1 | TMBIM4 |
| AKR1C3 | CEBPG | G6PDX | KIM-1 | NCF2 | SAT2 | TNFAIP3 |
| ALB | CFTR | GABARAPL1 | KLHL24 | NCOA4 | SCD | TP53 |
| ALOX12 | CHAC1 | GABARAPL2 | KRAS | NF2 | SCP2 | TP63 |
| ALOX12B | CHMP5 | GABPB1 | LAMP2 | NFE2L2 | SELENOS | TRIB3 |
| ALOX15 | CHMP6 | GCH1 | LINC00336 | NFS1 | SESN2 | TSC22D3 |
| ALOX15B | CISD1 | GCLC | LINC00472 | NGB | SETD1B | TUBE1 |
| ALOX5 | CISD2 | GCLM | LOC284561 | NNMT | SIRT1 | TXNIP |
| ALOXE3 | CP | GDF15 | LOC390705 | NOS2 | SLC11A2 | TXNRD1 |
| ANGPTL7 | CRYAB | GLS2 | LONP1 | NOX1 | SLC1A4 | UBC |
| ANO6 | CS | GLUT13 | LOX | NOX3 | SLC1A5 | ULK1 |
| ARNTL | CXCL2 | GOT1 | LPCAT3 | NOX4 | SLC2A1 | ULK2 |
| ARRDC3 | CYBB | GPT2 | LPIN1 | NOX5 | SLC2A12 | VDAC2 |
| ASNS | DDIT3 | GPX2 | LURAP1L | NQO1 | SLC2A14 | VDAC3 |
| ATF3 | DDIT4 | GPX4 | MAFG | NRAS | SLC2A3 | VEGFA |
| ATF4 | DNAJB6 | GSS | MAP1LC3A | OTUB1 | SLC2A6 | VLDLR |
| ATG13 | DPP4 | HAMP | MAP1LC3B | OXSR1 | SLC2A8 | WIPI1 |
| ATG16L1 | DRD4 | HBA1 | MAP1LC3C | PANX1 | SLC38A1 | WIPI2 |
| ATG3 | DRD5 | HELLS | MAP3K5 | PCBP1 | SLC39A14 | XBP1 |
| ATG4D | DUOX1 | HERPUD1 | MAPK1 | PCBP2 | SLC39A8 | YWHAE |
| ATG5 | DUOX2 | HIC1 | MAPK14 | PCK2 | SLC3A2 | YY1AP1 |
| ATG7 | DUSP1 | HIF1A | MAPK3 | PEBP1 | SLC40A1 | ZEB1 |
| ATM | EGFR | HILPDA | MAPK8 | PGD | SLC7A11 | ZFP36 |
| ATP5G3 | EGLN1 | HMGB1 | MAPK9 | PHKG2 | SLC7A5 | ZFP69B |
| ATP5MC3 | EGLN2 | HMGCR | MDM2 | PIK3CA | SNORA16A | ZNF419 |
| ATP6V1G2 | EIF2AK4 | HMOX1 | MIF | PLIN2 | SNX4 |  |
| AURKA | EIF2S1 | HNF4A | MIOX | PLIN4 | SOCS1 |  |
| BACH1 | ELAVL1 | HRAS | MIR137 | PML | SP1 |  |
| BAP1 | EMC2 | HSBP1 | MIR17 | PRDX1 | SQLE |  |
