# Supplementary material for: Machine learning reveals ferroptosis features and a novel ferroptosis classifier in patients with sepsis
Source: Immun Inflamm Dis. 2024 May 23;12(5):e1279. doi: 10.1002/iid3.1279 (PMC11112629; doi:10.1002/iid3.1279)
Supplement: Supplementary file 2 — Supporting information. [file IID3-12-e1279-s003.docx]

| **Table S2. The detailed features of each algorithm** | | | |
| --- | --- | --- | --- |
| **LASSO** | **RFB** | **SVM** | **XGB** |
| ATG16L1 | ATG16L1 | ATG16L1 | ATG16L1 |
| SRC | PML | SIRT1 | SIRT1 |
|  | VDAC3 | PML | PML |
|  | SRC | G3BP1 | G3BP1 |
|  |  | NRAS | NRAS |
|  |  | SRC | SRC |
|  |  | ANO6 | ANO6 |
|  |  | XBP1 | XBP1 |
|  |  | VDAC3 | VDAC3 |
|  |  | ELAVL1 | ELAVL1 |
|  |  | SLC2A6 | SLC2A6 |
|  |  | SLC3A2 | SLC3A2 |
|  |  | VDAC2 | VDAC2 |
