# Supplementary material for: Machine learning reveals ferroptosis features and a novel ferroptosis classifier in patients with sepsis
Source: Immun Inflamm Dis. 2024 May 23;12(5):e1279. doi: 10.1002/iid3.1279 (PMC11112629; doi:10.1002/iid3.1279)
Supplement: Supplementary file 3 — Supporting information. [file IID3-12-e1279-s002.docx]

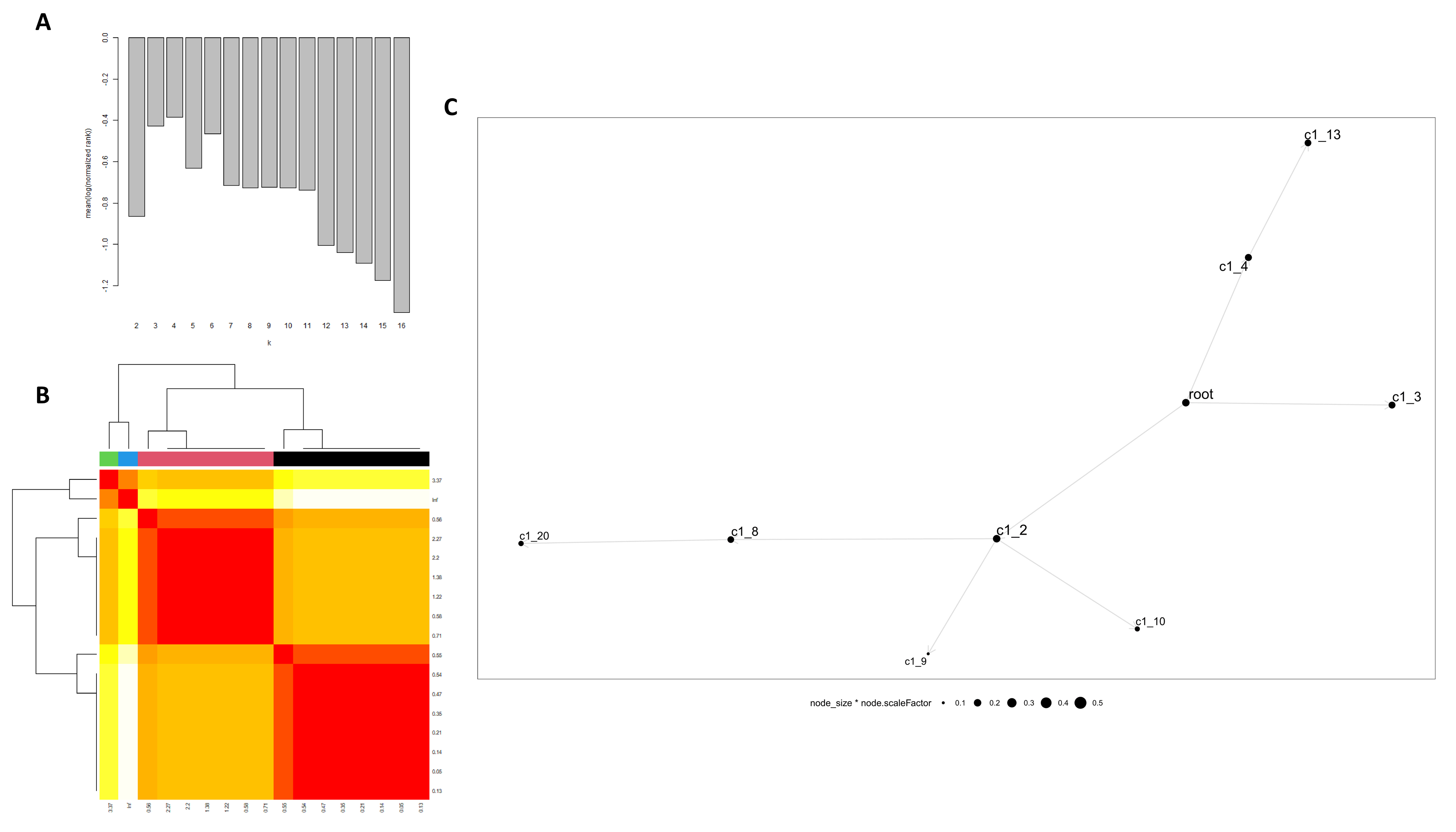


**Figure S1. Construction of MEGENA network**. **(A)** The rank chart showed the best K value = 4. **(B)** Construction of the module correlation heat map according to k = 4. **(C)** The branch tree constructed based on k = 4. Eight gene modules were obtained.


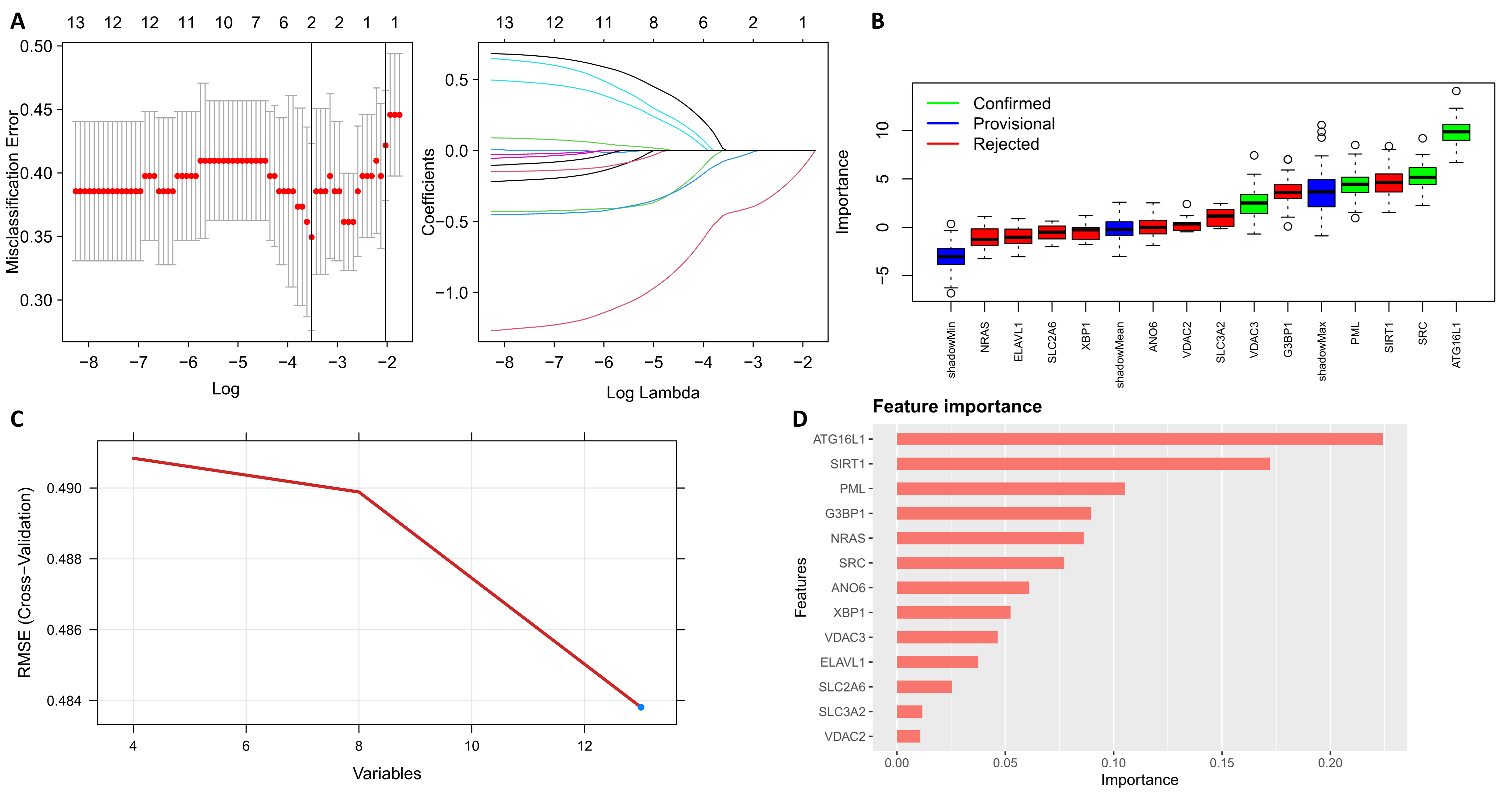


**Figure S2. Screening important death features through machine learning algorithms.** **(A)** Two ferroptosis characteristics according to the minimum lambda screening were obtained using the LASSO algorithm. **(B)** Four ferroptosis characteristics were screened using Random Forest Boruta. **(C)** The 13 ferroptosis features were screened using the SVM algorithm. **(D)** The 13 ferroptosis characteristics were screened using the XGBoost algorithm.

**
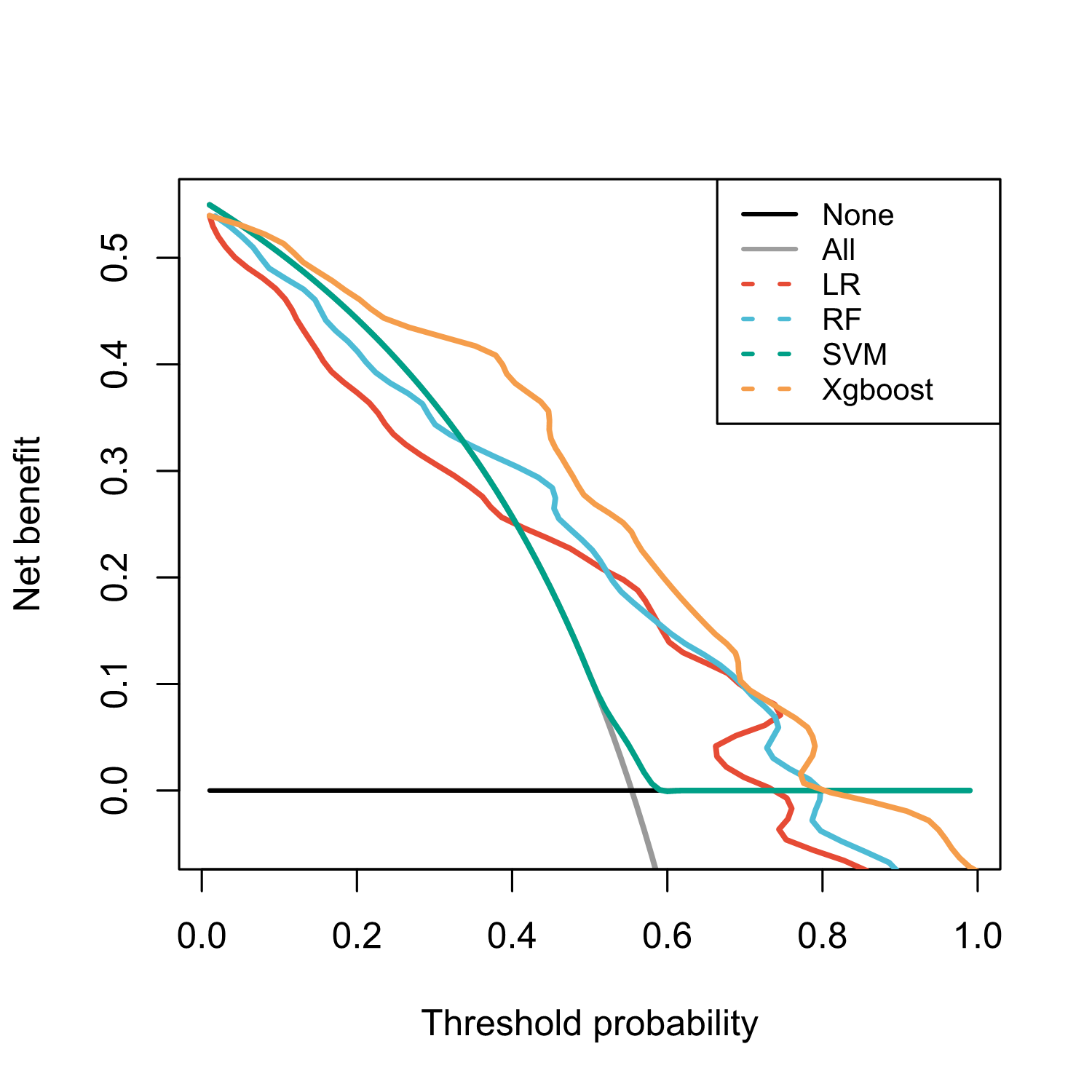
**

**Figure S3.** Decision curves were used to evaluate the benefits of the model, and the Xgboost model performs the best.
